# Enzyme-assisted extraction of leaf proteins: efficiency, functionality, and structural insights^☆^

**DOI:** 10.1016/j.fochx.2025.103181

**Published:** 2025-10-17

**Authors:** Ankita Sharma, Shalini Sharma, Godasritha Ramaraju, Prasad Rasane, Sezai Ercisli, Jyoti Singh

**Affiliations:** a Department of Food Technology and Nutrition, School of Agriculture, Lovely Professional University, Phagwara, Punjab 144411, India; b Department of Plant Pathology, Dr. Yashwant Singh Parmar University of Horticulture and Forestry, Nauni 173230, Solan, India; c Department of Horticulture, Faculty of Agriculture, Ataturk University, 25240 Erzurum, Türkiye; d HGF Agro, ATA Teknokent, Erzurum, Türkiye

**Keywords:** Enzyme-assisted extraction, Functional properties, Leaf protein, Process optimisation, Protein extraction, Protein characterisation

## Abstract

Leaf proteins are gaining popularity for their balanced amino acid composition and reduced environmental impact. Enzyme-assisted extraction (EAE) employs enzymes such as proteases, xylanases, β-glucosidases, pectinases, and alpha-amylases to degrade plant cell walls and facilitate release of target compounds. This review examines the role of EAE in green leaf protein extraction, identifies optimal process parameters, and evaluates the impact of EAE on the characterisation and functional properties of leaf proteins relative to conventional alkaline extraction. The improved functional properties of enzyme-extracted leaf proteins may enable their application as additives, emulsifiers, foaming agents, and nutritional enhancers in various food products.

## Highlights

• 80 % of the leaf proteins are present in the chloroplasts.
• Enzyme-assisted extraction (EAE) is a non-thermal, sustainable method.
• EAE enhances protein yield while preserving structural and nutritional integrity.
• Functional attributes like solubility, foaming, and emulsifying are improved by EAE.

## 1 Introduction

Ensuring a sustainable protein supply remains a crucial challenge for the global food system. Proteins are necessary for various processes, including development, growth, maintenance, maturation, lactation, and reproduction. They also help protect the body from diseases such as cancer when present in safe amounts. A protein deficiency negatively impacts both humans and animals. According to the World Health Organisation (WHO), in 2022, approximately 149.2 million children under five experienced stunting, and around 45.4 million deaths were linked to protein deficiency (Javed et al., 2023). The global population is expected to reach about 10 billion within the next three decades. On the other hand, the projected demand for food proteins is also set to increase by 76 % by 2050, placing significant pressure on current food systems. Protein consumption patterns reveal stark global disparities. Developed countries mainly consume animal-based proteins, as shown by the National Health and Nutrition Examination Survey (2015–2018), which reported a U.S. animal-to-plant protein consumption ratio of 2.1:1 (54.8 g/d animal protein, 25.8 g/d plant protein). However, the trend is opposite in many developing countries; for instance, Bangladesh consumes 80 % of the protein from plant sources only (Lolli et al., 2023; Messina et al., 2023). This disparity highlights a broader trend: some regions experience protein overconsumption, while others suffer from underconsumption. For example, the average European protein intake exceeds recommended levels by about 70 % (Aschemann-Witzel et al., 2021).

Animal proteins have good digestibility, a balanced essential amino acids content, and beneficial functional properties such as foaming, gelling agents, and emulsifiers in the food industry. However, their production requires large areas of land and water volumes and leads to the release of greenhouse gases (GHGs). Excessive consumption is also associated with negative effects on liver and kidney functions, calcium balance, and an increased risk of cancer, as well as bone and coronary artery diseases (Javed et al., 2023; Ortega et al., 2024). These challenges highlight the urgent need for sustainable, affordable, and nutritionally valuable alternatives to animal proteins. This review emphasises the growing importance of leaf proteins as an alternative to animal-derived sources, considering the rising demand for eco-friendly and nutritionally balanced food options. The per-hectare yield of leaf protein is estimated to be about four times higher than that of seed proteins annually (Kaur & Bhatia, 2023). Perennial leafy legumes, unlike annual crops such as wheat, soybeans, and maize, can utilise sunlight for most of the year and produce between 1000 and 3000 kg of crude protein per hectare (Møller et al., 2021). Globally, plant leaves are a significant source of protein, with approximately 80 % of proteins found in chloroplasts. Half of these proteins are soluble in the stroma, while the rest are part of the thylakoid membranes (Santamaría-Fernández & Lübeck, 2020). Furthermore, green leaf proteins offer several advantages, including reducing the harmful impacts of livestock farming, addressing ethical concerns related to animal welfare in the production of eggs, milk, and meat, and providing alternative protein sources for vegans. Although plant leaves are often discarded during processing, they can now be utilised for their protein content and can be converted into value-added food ingredients. Leaves such as tea, alfalfa, amaranth, olive, duckweed, radish, cabbage, sugar beet, *Moringa*, spinach, and cassava show promising protein contents (Hadidi et al., 2024). During plant growth, proteins serve as primary metabolites, and their levels typically decline as the plant matures. For example, in rapeseed stems and leaves, protein content decreased by approximately 35.6 % as the plant matured (Yu et al., 2015).

**Fig. 1:**
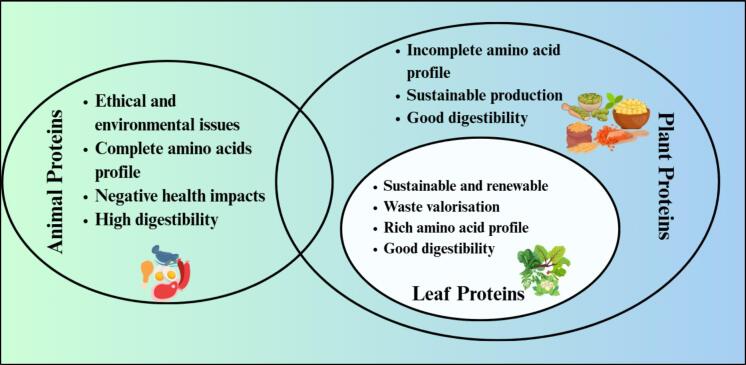
Comparative overview of animal, plant, and leaf proteins.

Although leaf proteins are abundant and sustainable, their poor extractability and functional limitations call for innovative green recovery methods like EAE. Fig. 1 provides a comparative overview of animal, plant, and leaf proteins. EAE is recognised as a sustainable and efficient method for protein recovery, utilising specific enzymes to break down complex biomolecules, including proteins and cell walls (Patil et al., 2025). Unlike other extraction methods, EAE is well-studied and scalable at an industrial level for the utilisation of by-products, such as in biorefineries, animal feed, and food industries (Kaur & Bhatia, 2023). EAE has been used to extract protein from almond cake (Souza et al., 2019), soybeans (De Moura et al., 2011), sacha inchi kernel cake (Chirinos et al., 2017), olive pulp and stone (Vergara-Barberán et al., 2014), olive leaves (Vergara-Barberán et al., 2015), among others. This review critically assesses EAE as a sustainable approach for leaf protein extraction, focusing on its ability to increase protein yield and functional properties, and evaluating its scalability for industrial application compared to conventional methods.

## 2 Global scenario of protein utilisation

Proteins are macronutrients that serve as a source of energy, nitrogen, and essential amino acids, and they significantly influence the functional, physicochemical, and organoleptic properties of foods (Chirinos et al., 2017). Around 2 billion individuals are estimated to be nutrient-deficient, which indicates hidden hunger. Inadequate quantity, poor quality, and unsafe food lead to malnutrition and adverse health outcomes. Moreover, a diet lacking in protein is considered the main cause of malnutrition (Kumar et al., 2021). By 2030, the global undernourished population is projected to increase from 688 million to 841 million. Although animal-derived protein is a resource-intensive and inefficient method of food production based on nitrogen utilisation, it still makes up 18 % of the world's protein supply (Piercy et al., 2023). Jiang et al. (2023) projected that by 2044, Asia and Africa will experience a significant increase in protein-energy malnutrition. Globally, the number of cases is expected to rise from 147,672,757 in 2019 to over 160 million in 2044. This disparity is mainly due to the dense population and limited land resources in these areas. Bayati et al. (2025) highlighted that socioeconomic factors such as living conditions, maternal occupation, cultural influences, prenatal care, and parental education significantly impact the prevalence of protein-energy malnutrition. Low-income regions face challenges related to access and quality of protein, while high-income areas expand the plant-protein market through increased consumer awareness and corporate strategies. The close ties of some governments with transnational agri-food corporations (TNCs) also influence protein consumption trends. Recently, many major companies like Cargill, JBS, McDonald's, Nestlé, Kerry Group, etc., have invested heavily in acquiring plant-based substitutes or developing their own. The reason for this shift is evident. The meat substitute market is expected to reach US$12 billion annually by 2025 and US$17 billion by 2027, with a projected annual growth rate of 15–18 % from 2020 to 2025 (Béné & Lundy, 2023). These trends are driven by rising consumer awareness of sustainability and the environmental impacts of the food system, prompting companies to innovate and optimise processes related to energy use, carbon footprint, and waste valorisation (Lolli et al., 2023).

The trends in health and fitness, as well as the growing popularity of vegetarianism and veganism, are some of the reasons behind the increasing demand for plant-based protein. In 2022, Markets and Markets (MAM) and the 10.13039/501100000046National Research Council Canada (NRCC) reported that the global plant-based protein market alone reached $10.8 billion, supported by a CAGR of 6.7 %, and is projected to reach $17.4 billion by 2027 (Yeasmen & Orsat, 2024). Due to the variability in environmental conditions, including climate, soil, socioeconomic factors, and cultural differences, sustainable protein production varies significantly worldwide. In some regions, the production of plant-based proteins has the potential to increase substantially. In others, this production will be limited and yields non-human edible, low-quality proteins suitable only for livestock (Leinonen et al., 2020). The United Nations Food System Summit in 2021 and the 2030 Agenda have called for urgent reform of global food systems to meet sustainable development goals. Concerns related to animal protein include its inability to meet rising population demands, links to coronary heart disease and obesity, environmental impacts from GHG emissions, and ethical issues surrounding animal welfare (Zhang et al., 2024). These issues have significantly increased the demand for plant-based proteins. Aschemann-Witzel et al. (2021) reviewed the main reasons for this change. In various countries, nutritional guidelines are being suggested for revision with environmental issues in mind. For example, Sweden implemented such changes in 2015. These types of guidelines promote plant-based diets and discourage meat and dairy consumption, thereby influencing consumer demand. Additionally, governments and organisations have set climate targets; for instance, Denmark's food and agricultural sector aims to achieve climate neutrality by 2050, which is expected to further boost demand for plant-based foods and meat alternatives. In the food sector, protein plays a vital role due to its various functionalities, such as water retention capacity, solubility, colour, oil retention capacity, texture, viscosity, emulsification, and foaming index. These attributes vary with factors like pH, protein concentration, and temperature. The ability of plant-based proteins to generate foam and facilitate emulsification in products such as cakes, whipped toppings, confectionery items and creams makes them a viable alternative (Akyüz et al., 2024).

## 3 Conventional methods of protein extraction

Several components are present in the plant cell wall besides soluble compounds that are difficult to extract via conventional methods. Polysaccharides such as starch, hemicellulose, and pectin are abundant in the cell wall, reducing the efficiency of extraction with conventional techniques (Nadar et al., 2018). Conventional techniques include wet extraction methods (alkaline, salt and acid extraction) and dry fractionation methods to extract plant proteins (Tang et al., 2024). Alkaline extraction is the predominant method for extracting proteins from plant and seed sources. This technique disrupts the cell wall by partially removing lignin and modifying the chemical composition and structure of cellulose (Perović et al., 2020). Despite the improved extraction efficiency of alkaline extraction, the drawbacks associated with the process limit its applications. For instance, extraction is known to significantly affect the digestibility of protein while also disrupting the structures of amino acids such as lysine and cysteine (Kumar et al., 2021). The process is associated with the conversion of amino acids like serine and cysteine residues, which generate compounds like nephrotoxic lysinoalanine that can further lead to the impairment in the bioactivity of protein (Hewage et al., 2022). Strong alkaline conditions alter protein structure and functionality by inducing denaturation, aggregation, and exposure of nonpolar and sulfhydryl groups. These changes increase hydrophobic interactions and promote disulfide bond formation, thereby reducing protein solubility. Furthermore, protein degradation processes, including hydrolysis and Maillard reactions, are likely to occur at pH values above 10 (Furia et al., 2025). Moreover, extreme alkaline conditions promote the oxidation of phenols, resulting in the formation of highly reactive quinone molecules. These quinones covalently interact with proteins, which leads to the formation of protein aggregates. Covalent protein-phenol interactions can also alter protein structure and frequently reduce protein surface hydrophobicity (Yang & Sagis, 2021). These modifications decrease protein digestibility and bioavailability, reducing its suitability for human food applications.

**Fig. 2:**
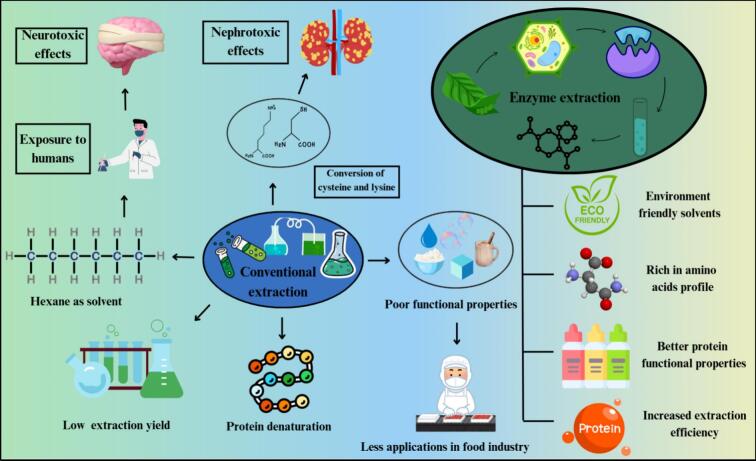
Advantages of enzyme extraction over conventional extraction techniques

Acid extraction enhances protein solubility at acidic pH levels below the isoelectric point. However, it is less effective because of reduced cell wall degradation and low solubility near the isoelectric point (Rahman & Lamsal, 2021). Neutral pH salt solutions, including sodium chloride, calcium chloride, and potassium chloride, serve as standard reagents for salt extraction. Salt extraction operates by precipitating proteins through salinisation and salting-out, followed by removal of insoluble material via sedimentation, decantation, sieving, and centrifugation. Dry fractionation is a mechanical process that separates proteins from starch and additional cellular components. Acid extraction methods are inadequate because they negatively impact the solubility and gelation properties of extracted proteins. Dry fractionation techniques, including air separation, result in residual proteins within starch fractions, thereby reducing overall protein yield. Many conventional extraction processes require large volumes of solvents, which are highly volatile, flammable, and toxic. These hazardous solvents may persist on proteins and pose health risks towards humans (Tang et al., 2024). The solvent extraction method can achieve higher extraction rates; however, the use of flammable solvents like hexane raises safety and environmental concerns (de Almeida et al., 2019). n-hexane is regarded as a popular solvent in the chemical and food industries; however, it is explosive, highly flammable, and can cause mild central nervous effects in humans (Liu et al., 2016). These environmental and health risks highlight the need for safer, enzyme- or green-technology-based alternatives as shown in Fig. 2**.**

## 4 Enzyme-assisted extraction for protein extraction

Proteins in plant tissues are structurally bound to complex matrices like fats, carbohydrates, fibre, and polyphenols; therefore, an efficient extraction method is required to break these bonds, dissolve the structural membranes and cell walls, and effectively release the proteins (Ampofo & Ngadi, 2022). EAE works on the principle of hydrolysing the plant cell wall using specific enzymes as catalysts under optimal conditions to release intracellular components. The plant cell wall binds to the enzyme's active site. This binding induces a conformational change in the enzyme, facilitating effective interaction with the substrate. The resulting structural changes in the enzyme promote the cleavage of cell wall bonds, thereby releasing the active constituents (Nadar et al., 2018). EAE uses enzymes such as xylanases, cellulases, proteases, hemicellulases, β-glucosidases, pectinases, and α-amylases to extract target constituents from plant cells. The process efficiency depends on factors like enzyme type and concentration, extraction temperature, pH, and others (Amulya & Ul Islam, 2023).

EAE offers several advantages over conventional extraction. By breaking down cell walls into smaller sizes, carbohydrase cocktails containing pectinase and cellulase improve liquid diffusion inside cells and can increase protein yield. However, protease is considered better than carbohydrase, but its higher price limits its applications in the industry (Rahman & Lamsal, 2021). Various enzymes, such as carbohydrases, proteases, and complexes like Papain, Flavourzyme, Celluclast, Alcalase, amyloglucosidase, Energex, Viscozyme L, α-amylase, and hemicellulase, have shown potential in extracting proteins from vegetable sources (Miranda et al., 2022). Studies have reported significant improvements in protein yield, content, and digestibility when using EAE. Alpha-amylase hydrolyses the starch component bound to protein, thus enhancing protein accessibility and extraction. In radish leaf protein concentrate (RLPC), alpha-amylase extraction yielded high protein content of 89.41 % and a yield of 9.56 %, with an in-vitro digestibility of 92.17 %, as shown in Table 1 (Kaur & Bhatia, 2023). In contrast, alkaline extraction resulted in slightly higher protein yield (12.12 %) but lower protein content (87.64 %), with comparable digestibility (93.51 %) (Kaur & Bhatia, 2022). Wang et al. (2025) also reported that xylanase/cellulase-assisted extraction increased RLPC yield by up to 208.59 %. Enzymatic cocktails can further improve results; for example, Viscozyme L achieved a protein yield of 14.2 %, a protein content of 55.7 %, and superior digestibility of 99.8 % for *Moringa Oleifera* defatted leaf protein concentrate (MODLPC). Protein bioaccessibility values for MODLPC were 556.1 mg/g. Anti-nutritional components like tannins, phytic acids, and oxalic acids found in green leaf proteins can limit efficient protein utilisation, absorption, and digestion, leading to decreased nutritional quality and bioavailability. However, removing antinutritional factors during extraction may enhance protein digestibility (Benhammouche et al., 2021). In addition to increasing yield, enzymes facilitate the production of protein fractions and isolates with reduced environmental impacts by lowering the need for extensive milling or solvents and improving the yield and functional properties of the extracted proteins (Gouseti et al., 2022). Despite its advantages, EAE has several drawbacks as well. There is a need for sample-specific enzyme selection, a lengthy optimisation process to achieve high recovery yields with minimal enzyme use, variations in enzyme activity between batches, and extra steps for enzyme inactivation or separation (Kleekayai et al., 2023). Over-hydrolysis poses another risk, as it can degrade proteins and reduce their extractability (Patil et al., 2025).

**Table 1:** Optimal conditions for enzyme extraction of leaf proteins, along with achieved protein yield and content.

Raw material	Enzymes used	Optimal conditions	Protein yield (%)	Protein content (%)	References
Cauliflower leaf protein	Viscozyme® L Vinozyme®	pH 4.5, 10 h, 35 °C, (E/S 0.2–4.8 %)	14.90	48.23–77.27	Sedlar et al. (2025)
Broccoli leaf protein			29.88	52.03–84.66	
Mallow leaf protein	Pectinex UF	pH 5.6, 52.5 °C, 75 min, 7 % enzyme concentration	82.2	40–61.73	Ersus and Akyüz (2023)
	Pectinex Ultra SP-L	pH 7, 48 °C, 75.5 min and 7 % enzyme concentration	77.9		
Sugar beet leaf protein	Pectinex Ultra SP-L	54.25 °C, 81.35 min, solvent/solid ratio of 27.65 mL/g	55.15	69.08	Akyüz and Ersus (2021)
*Raphanus sativus* L. leaf protein	Alpha-amylase	42.8 °C, 4.44 h, 18,446 U enzyme concentration	9.56	89.41	Kaur and Bhatia (2023)
*Moringa Oleifera* defatted leaf protein	Viscozyme L.	30 °C, 30 min and 60 FBG for enzyme concentration	14.2	55.7	Benhammouche et al. (2021)

E/S: Enzyme substrate concentration; FBG: Fungal Beta-Glucanase Units.

### 4.1 Optimal extraction parameters

The functional properties of proteins, such as gelation, solubility, emulsification, foaming, etc., determine their applications in the food sector. To preserve the composition and conformation of the protein, proteins must be isolated under mild processing conditions that can minimise structural damage (Nadar et al., 2018). Key factors affecting the efficiency of EAE include reaction time, pH, temperature, enzyme, and substrate concentration. Optimising these factors is important to acquire enzymatic treatment while remaining cost-effective. However, to optimise these reaction conditions, extensive knowledge of the specific enzyme and substrate properties is necessary, as interactions between enzymes and plant matrices can vary significantly (Gouseti et al., 2022). EAE can be optimised to maximise protein recovery while minimising enzyme usage. Statistical tools such as design of experiments (DOE), Box-Behnken design (BBD), factorial design, response surface methodology (RSM), and central composite design are used for the optimisation process. These tools systematically evaluate the individual and interaction effects of processing parameters like temperature, pH, enzyme concentration, and time on protein yield (Kleekayai et al., 2023).

#### 4.1.1 Pretreatment of biomass and particle size

In the current literature, leaf samples for protein extraction are prepared by washing, drying, and pounding the leaves into powder; however, such minimal preparation may not be sufficient, as antinutritional components, high moisture content, and temperature conditions can significantly affect protein yield and functionality. Pretreatment of biomass is an effective step to reduce moisture content, preserve heat-sensitive components, and enhance enzymatic efficiency during extraction. Among pretreatment methods, soaking has been shown to effectively reduce antinutrient content. A study conducted by Sahoo et al. (2024) found that soaking decreased phytate content more effectively compared to steaming and boiling, reflecting the heat-stable nature of phytic acid. However, prolonged soaking resulted in the reabsorption of phytates and emphasised the need for process optimisation. Soaking also reduced oxalate and tannin content by 28.57 % and 87.36 %, respectively, while saponin and trypsin inhibitor activity showed reductions of 85.36 % and 68.33 %, respectively. A 59.23 % decrease in α-amylase inhibition activity was observed, indicating improvements in digestibility. Another emerging pretreatment is fermentation, which can be used to lower antinutrient content and increase protein bioavailability. Terefe et al. (2022) reported that fermentation increased the protein content of cassava leaves from 17.79 % to 24.0 %, and this increase could be attributed to the formation of non-protein nitrogen compounds like amines, peptides, ammonia, and amino acids. Additionally, this effect may also result from the production of specific extracellular enzymes, which are protein-based catalysts, by fermenting microorganisms. Examples of these enzymes include amylase and linamarase, both produced by fermenting microorganisms. Amylase facilitates the degradation of starch molecules in cassava leaves, while linamarase is involved in the detoxification of cyanide compounds. The study showed a 97.17 % decrease in cyanide content. Similarly, oxalates and tannin content also decreased significantly through enzymatic hydrolysis, along with the production of tannase enzyme in the fermented product. Hence, while soaking is a simple, effective process to reduce phytates and tannins, fermentation offers broader detoxification and protein enhancement, making it more suitable for leaves with high cyanide content.

Ultrasound pretreatment has also been explored in the literature and has been reported to enhance the enzymatic hydrolysis of proteins. This pretreatment is cost-effective, and high-intensity ultrasound pretreatment can modify protein conformation by affecting hydrogen bonds and hydrophobic interactions, disrupting the quaternary and/or tertiary structure of globular proteins, thereby exposing more hydrolysis sites for enzymatic accessibility (Chen et al., 2011). Freeze drying or lyophilisation is a mild process, and its low temperature and pressure conditions prevent Maillard reactions and enzymatic browning. However, it is an energy-intensive process; therefore, an alternative approach is grinding samples under liquid nitrogen before drying. This method involves grinding the food matrix into small particles followed by freeze-drying (Ebrahimi et al., 2021), minimising protein denaturation and enhancing extraction efficiency. Another factor influencing extraction yield is the particle size of the plant biomass; smaller particles provide better results due to increased surface accessibility for enzymes (Kleekayai et al., 2023).

#### 4.1.2 Selection of enzymes

Enzyme selection is a vital step in maximising protein extraction efficiency. Carbohydrases impact protein extractability by destroying the cell wall. The protease enzyme increases protein yield by acting on the protein-polysaccharide matrix and releasing protein. Proteins extracted by enzymes have low molecular weight and higher solubility (Navaf et al., 2023). Plant cell walls, made up of macromolecules like pectin, cellulose, and hemicellulose, serve as barriers that can be broken down by specific enzymes such as pectinases and carbohydrases like cellulase. Proteolytic enzymes like Alcalase® and papain are effective in increasing extraction yield by separating proteins from the surrounding polysaccharide matrix (Gouseti et al., 2022; Hadidi et al., 2024; Vergara-Barberán et al., 2015). Partial protein hydrolysis by proteases enhances solubility by releasing hydrophilic components; however, it can partly damage proteins, reducing their functionality. Additionally, prolonged exposure to high temperature or pH may modify amino acids like cysteine and lysine, negatively impacting the quality and digestibility of the extracted proteins (Heppner & Livney, 2023). Amylase also play a role by hydrolysing the protein-bound starch components and breaking down cell wall polysaccharides, providing an environmentally friendly method to improve protein accessibility. Various studies emphasise the significance of enzyme type and dosage. For example, α-amylase achieved the highest protein extraction compared to xylanase and protease (Kaur & Bhatia, 2023). In mallow leaf (*Malva sylvestris* L.), Pectinex-UF produced 8.9 times higher yield than Pectinex Ultra SP-L (Ersus & Akyüz, 2023). In sugar beet leaves, Pectinex Ultra SP-L increased extraction efficiency by 43.27 %, with a yield of 79.01 %, due to its pectolytic activity that breaks glycolytic bonds in the pectin chain (Akyüz & Ersus, 2021). Enzyme loading also plays an important role; for instance, yields increase with the enzyme-to-substrate ratio, as seen in the study by Sedlar et al. (2025). However, as Vergara-Barberán et al. (2014) noted, higher enzyme loading can reduce protein content, possibly because of turbidity in the protein extract.

#### 4.1.3 Temperature and time

Temperature and time are crucial factors influencing protein extraction during EAE. Generally, an extraction time of less than 100 min and a temperature range of 50–60 °C produce favourable results (Sari et al., 2015). Naseri et al. (2020) reported that protein extraction efficiency increased from 72.4 to 76.4 % at 30 °C to 83.8–84.4 % at 60 °C. A similar trend was noted for RLPC, where yields dropped significantly after 45 °C (Kaur & Bhatia, 2023). Vergara-Barberán et al. (2015) optimised protein extraction from olive leaves at 55 °C for 15 min; increasing the temperature reduced yields due to protein degradation, while extending the extraction time had a minor impact. These results can be explained by thermal denaturation, where heating proteins above a critical temperature destabilises electrostatic interactions, hydrogen bonds, and van der Waals forces, while hydrophobic interactions are stabilised, ultimately causing protein unfolding. Factors such as inorganic salts, ionic strength, pH, solvents, and others also influence protein thermal stability (Pathania et al., 2018).

De Almeida et al. (2019) optimised enzyme-assisted aqueous extraction of almond flour, achieving a protein yield of 64–79 % at pH 9 and 50 °C for 60 min. This study noted that although enzyme use did not significantly increase extraction efficiency, it enhanced the functionality of the extracted protein. Table 1 highlights the optimal conditions for the EAE of various leaf proteins, including their protein yield and content. Overall, while higher temperatures can accelerate extraction as reported by Zhang et al. (2014), where yields increased to approximately 95 % at 95 °C in 4 h for alkaline extraction, higher temperatures also promote coagulation, hydrolysis, denaturation, and amino acid racemisation, ultimately reducing protein quality. Thus, moderate conditions are preferred to balance yield, stability, and functionality of the protein.

### 4.2 pH and solvent selection

Selecting a solvent for enzymatic reactions is a crucial step. While aqueous media remain the standard for industrial use, alternative systems like biphasic mixtures, supercritical fluids, reversed micelles, or enzyme suspensions in anhydrous solvents are being explored, especially for reactions involving poorly water-soluble compounds. Ionic liquids have also emerged as environmentally friendly solvents because of their ability to stabilise proteins, enhance enzyme resilience, and preserve functional properties at higher temperatures (Gouseti et al., 2022). Naseri et al. (2020) further highlighted that Alcalase® hydrolyses peptide bonds between amino acids, thereby increasing protein extraction. Additionally, combining protease enzymes with alkaline extraction was found to be more efficient than using polysaccharide-degrading enzymes with alkaline extraction. Moreover, pH influences protein extraction by altering the properties of the cell wall and proteins (Sari et al., 2015). Yu et al. (2015) emphasised that at neutral pH, experiments can yield relatively high protein levels and improved solubility.

## 5 Characterisation of proteins

Leaf protein is a concentrate made from the stems and leaves of a fresh green plant by squeezing the juice, separating the protein, concentrating it, and drying it. Usually, the final protein yield is around 40–60 % of the total leaf protein mass (Cao et al., 2023). Nynäs et al. (2021) emphasised that the origin of leafy biomass significantly influences the protein fractionation outcome, challenging the idea that any green leafy biomass can serve as raw material in a general protein extraction process. Protein quality depends on the amino acid profile, which is crucial for the protein's role in processes like acting as a biocatalyst and a carrier for minerals and vitamins. Chromatography methods, such as High-performance liquid chromatography (HPLC), are used to purify proteins, while spectrometry methods like Liquid chromatography–mass spectrometry (LC/MS) measure the amino acids in the protein. Techniques like Sodium dodecyl-sulfate polyacrylamide gel electrophoresis (SDS-PAGE) and Two-dimensional gel electrophoresis (2-DE) identify protein components such as albumins, globulins, and prolamins based on their molecular weight. Scanning Electron Microscopy (SEM) analyses the structural morphology, while methods like Differential Scanning Calorimetry (DSC) and Isothermal Titration Calorimetry (ITC) predict structural changes and assess the thermal properties of biomolecules (Kumar et al., 2022; Rao & Poonia, 2023).

FTIR analysis is an important technique used for understanding the molecular structure of proteins and their composition. According to frequency-decreasing order, nine amide bands, including A, B and I-VII, are generated by peptides and proteins, where amide I-III are mainly used to analyse the structure of proteins. The amide I band is seen in the region of 1600–1690 cm^−1^, and is reflected by the C=O stretching vibration that is a result of amide groups, which are weakly coupled with in-plane NH-bending and CN stretching vibrations. In addition, Amide *I* bands are indicative of β-sheet secondary structures in the region of 1615–1640 cm^−1^. The peaks within the 1480–1575 cm^−1^ region are attributed to amide band II, which is generally an out-of-phase combination of the NH bending and CN stretching vibrations, with minor contributions from CO in-plane bend as well as NC and CC stretching vibrations. The Amide III band is mainly noted in between 1229 and 1301 cm^−1^. It is primarily an in-phase combination of NH bending and CN stretching vibrations with additional minor contributions from CO in-phase bending and CC stretching vibrations (Kaur & Bhatia, 2023; Wei et al., 2024).

In RLPC, the peaks at 1617.67 and 1636.38 cm^−1^ corresponded to the Amide I band and β-sheets as secondary structures. The 3237 cm^−1^ band, in particular, showed strong intramolecular C—O…N—H H-bonds compatible with α helical conformation, and the bands at 3414 and 3473 cm^−1^ correspond to amide A and amide B linkages, primarily resulting from intermolecularly hydrogen-bonded NH groups (Kaur & Bhatia, 2023). In contrast, the protein concentrates from cauliflower and broccoli leaves (CLP and BLP) exhibited peaks at 3270 cm^−1^, 2920 cm^−1^, 1632 cm^−1^, 1516 cm^−1^, and 1218 cm^−1^. The concentrate shows amide I–III regions, while the region between 3000 and 3700 cm^−1^ is linked with the vibrations of amino groups and the hydrogen bonding in the polypeptide. The 2800–3000 cm^−1^ region showed adsorption of C—H bond stretching. Comparatively, RLPC demonstrated more organised β-sheet enrichment, whereas BLP and CLP display a broader distribution of amide bands, indicating structural diversity and differences in hydrogen bonding. It is also observed that an increase in the enzyme concentration enhanced the absorption intensity of all characteristic amide peaks. However, at a low E/S ratio (0.2 %), absorption was observed to be lower than in the control. At the higher E/S ratio of 4.8 %, both BLP and CLP exhibited the highest absorption values, consistent with their protein content. Notably, despite its higher protein content, BLP showed weaker FTIR absorbance than CLP, suggesting that the presence of non-protein matrix components may interfere with amide bond signals and decrease spectral intensity (Sedlar et al., 2025).

SDS-PAGE is a common technique where different stains are used to visualise proteins to analyse their molecular weight. Vergara-Barberán et al. (2015) reported different bands like 63 kDa (oleuropein β-glucosidase), 110 kDa and 29 kDa in the protein extracts of olive leaves depending on the genetic variety; however, the 55 kDa band remained common in all genetic varieties. Similarly, broccoli and cauliflower protein concentrate showed prominent protein bands at 20 kDa corresponding to globulin fractions, while 14 kDa signified the albumin fraction of proteins. Other detected protein bands corresponded to molecular weights of about 25 kDa–45 kDa (Sedlar et al., 2025). Unlike cruciferous leaves, RLPC bands showed broader molecular weight distribution, ranging from 35 to 92 kDa. Three distinct bands at 45, 50 and 57 kDa represented the distribution of albumin polypeptides, while bands between 40 and 66 kDa corresponded to glutelins. A characteristic smear of bands between 47 and 60 kDa referred to the polypeptide composition profile of globulins, and a slightly faint band at 47 kDa represented prolamins (Kaur & Bhatia, 2023). Alkaline-extracted RLPC displayed poor separation of bands, with albumins and prolamins spanning from 14 to 55 kDa, and gluteline and globulins observed to be smeared bands between 20 and 30 kDa. This is likely due to the limited solubility of these fractions in electrophoresis buffer or the heterogeneous nature of polypeptides in the RLPC and protein fractions (Kaur & Bhatia, 2022). Alkaline conditions may contribute to partial denaturation or aggregation. These results highlight that enzyme extraction methods produced well-defined, clearer bands for albumins, glutelin, and globulins, demonstrating better preservation of protein structural integrity compared to alkaline extraction. Comparative analysis reveals that RLPC exhibits greater protein heterogeneity, whereas CLP and BLP are characterised by the predominance of smaller peptide bands. Olive leaves showed greater variability between their genetic varieties, but the RuBisCo subunit remained constant throughout.

Microscopy techniques such as SEM, TEM, and AFM have been used to understand the morphological properties of different proteins obtained from plants while also providing an idea of the nutrient localisation and bio-accessibility. SEM, particularly, is a magnification technique for surface imaging which gives information about the morphology, particle size, structure and modification in plant proteins. This helps determine the role of proteins in the food industry. For instance, uniformity and a reduced particle size of proteins are associated with mechanically strong plant-protein-based packaging films (Kumar et al., 2022). Also, the structure and morphology of the protein concentrate are affected by various attractive and repulsive forces that are associated directly with the extraction conditions applied. The SEM images of enzyme-extracted freeze-dried RLPC showed a cloudy shape, consisting of irregularly formed networks and rough surfaces, which was suggested to have an impact on the oil retention and other emulsion properties. In addition, chemical modifications and pH changes during the extraction process might have contributed to the rough structure of proteins and surface depressions in the microstructures that can further affect the functional attributes of the protein concentrate (Kaur & Bhatia, 2023). Drying affects the SEM results of the protein. For instance, the mentioned study used freeze drying, also known as lyophilisation, which is used to form solid protein formulations. Freeze-dried process is a batch process and requires extra processing sometimes (e.g. milling) while also producing stress like cold denaturation that can further damage the protein structure (Mutukuri et al., 2020). Amino acids are known to be the essential building blocks of proteins and are known to have specific roles in metabolic pathways. These amino acids further synthesise neurotransmitters, signalling molecules, biomolecules, and hormones, and can directly and indirectly influence biochemical pathways at the cellular level for processes such as growth, maintenance, and metabolic activities. The structure of all amino acids possesses both carboxylic and amine functional groups. Except glycine, all amino acids have an asymmetric α‑carbon (chiral centre), which further results in the generation of D- and L-enantiomers. This distinctiveness in their side chains leads to their biochemical diversity due to the presence of acidic, basic, polar, and nonpolar natures (Kumar et al., 2022).

Threonine and valine were found to be abundant in most leaf proteins, with MODLPC showing 6.86 g/100 g and 5.31 g/100 g for threonine and valine, respectively, as shown in Table 2**.** In alkaline-extracted *Moringa* leaf protein, glutamic acid (16.80 g/100 g) and histidine (1.47 g/100 g) were higher than those of enzyme-extracted MODLPC (Javed et al., 2023). In sugarbeet leaf protein (SLP), serine was higher than other amino acids, indicating the variability based on the source of the leaf. However, SLP showed the lowest values for other amino acids compared to other leaf proteins, making it a poor source. Ornithine and taurine were only detected in mallow leaf protein (MLP) at low levels. The most abundant amino acid in enzyme-extracted CLP was aspartic acid (6.83 g/100 g), followed by leucine (4.15 g/100 g). Similar results were observed in enzyme-extracted BLP, with aspartic acid (6.19 g/100 g) and leucine (3.82 g/100 g) being the most abundant amino acids. The ratio of essential to non-essential amino acids was more than 0.6, exceeding recommended standard values of FAO/WHO that are in the range of 0.6–0.7 (Sedlar et al., 2025). Compared to other conventional techniques, EAE gives a better amino acid profile. Prolonged exposure to these alkaline conditions may also result in the denaturation and hydrolysis of the proteins with significant loss of essential amino acids such as lysine (Hadidi et al., 2024). Amino acids present in these leaves are essential for the human body. For example, phenylalanine plays an important role in the synthesis of other amino acids, while lysine is mainly utilised in the production of antibodies. Moreover, serine participates in the synthesis of purines and pyrimidines and glutamic acid is involved in the metabolism of carbohydrates and fats and is important for the functions of keratin, DNA and RNA (Ersus & Akyüz, 2023).

**Table 2:** Amino acid profile of various plant leaves extracted using enzyme-assisted extraction.

Amino acids	MLP	SLP	CLP	BLP	MODLPC	References
Essential amino acids (g/100 g)						
Methionine	0.21–0.46	0.07	1.16	0.85	1.44	(Akyüz & Ersus, 2021; Benhammouche et al., 2021; Ersus & Akyüz, 2023; Sedlar et al., 2025)
Lysine	0.71–1.51	0.09	3.11	2.94	5.05	
Phenylalanine	0.83–1.81	0.31	ND	ND	5.04	
Threonine	1.29–2.81	0.37	3.60	3.55	6.86	
Valine	0.80–1.62	0.35	3.87	3.60	5.31	
Leucine	1.03–2.34	0.29	4.15	3.82	5.89	
Isoleucine	0.54–1.31	0.31	3.40	3.41	3.61	
Non-essential amino acids (g/100 g)						
Arginine	0.48–1.01	0.15	3.13	2.96	9.42	
Histidine	0.28–0.83	0.02	1.18	1.26	1.37	
Tyrosine	1.12–2.29	0.26	4.26	3.29	3.94	
Alanine	0.95–2.18	0.25	3.06	2.76	2.26	
Aspartic acid	2.09–5.23	0.65	6.83	6.19	12.15	
Glutamic acid	1.23–2.59	0.33	3.52	3.56	10.82	
Glycine	1.02–2.29	0.23	3.46	3.19	2.33	
Proline	0.58–1.21	0.20	0.07	0.02	2.79	
Serine	1.37–2.77	4.87	3.12	2.88	5.90	
Cysteine	0.39–0.62	ND	0.13	0.26	8.29	
Ornithine	0.09–0.10	ND	ND	ND	ND	
Taurine	0.04–0.03	ND	ND	ND	ND	

ND: Not detected; MLP: Mallow leaf protein; SLP: Sugar-beet leaf protein; CLP: Cauliflower leaf protein; BLP: Broccoli leaf protein; MODLPC: *Moringa Oleifera* Defatted leaf protein concentrate.

## 6 Effect on the functional properties

Techno-functionality of proteins encompasses physicochemical properties that influence the texture, appearance, and stability of food products. Key properties include solubility, viscosity, foaming capacity, emulsifying ability, gelling ability, and fat absorption capacity (Pojić et al., 2018). Interactions with water, proteins, lipids, carbohydrates, and other compounds, as well as several factors, including pH, time, temperature, and ionic strength of the solvent, affect the techno-functional attributes of plant proteins. For instance, high temperature and pH can result in poor nutritional and functional attributes of the extracted protein isolate. The development of a dark colour in conventionally extracted proteins is attributed to the co-extraction of undesirable compounds. During alkaline extraction, polyphenols undergo oxidation to form o-dihydroxy or o-quinones structures. These reactive compounds can form covalent bonds with thiol or amino groups of proteins at high pH, or non-covalent hydrogen bonds at low pH, resulting in a dark green to brown colouration in protein ingredients. The presence of these compounds also impairs the functional properties of proteins (Hewage et al., 2022). Furthermore, protein characteristics such as structural configuration, transformation processes, proximate composition (including lipid, protein, carbohydrate, ash, and moisture content), amino acid profile (polar, nonpolar, sulphur-containing), and physicochemical parameters (molecular weight, size, intrinsic viscosity) exert significant effects on functional properties (Anoop et al., 2023).

Intrinsic factors such as composition of amino acids, hydrophobicity, and extrinsic factors including ionic strength, drying techniques, extraction methods, and pH affect the protein solubility. Amino acids are classified as polar (asparagine, cysteine, serine, methionine, tyrosine, threonine, glutamine), non-polar (alanine, glycine, proline, valine, leucine, isoleucine, phenylalanine, tryptophan), acidic (glutamic acid, aspartic acid) and basic (arginine, lysine, histidine). The polar and non-polar amino acids are hydrophilic and hydrophobic, respectively (Gao et al., 2024). Protein solubility further affects the emulsifying and foaming properties, thus eventually affecting the application of proteins in the food industry. The functional properties of various enzyme-extracted leaf proteins are stated in Table 3**.**

**Table 3:** Functional properties of various enzyme-extracted leaf proteins.

Raw material	Functional property	Value	Reference
Cauliflower leaf protein	Protein solubility	29.4 mg/mL at pH 11	Sedlar et al. (2025)
Broccoli leaf protein		36.4 mg/mL at pH 10	
Mallow leaf protein	Bulk density	92.59–435.70 kg/m^3^	Ersus and Akyüz (2023)
	Tapped density	318.51–870.27 kg/m^3^	
	Particle density	45.68–70.93 kg/m^3^	
	Carr index	1659.35–1926.64 %	
	Water activity	0.2–0.3	
	Protein solubility	94.4 % at pH 8.5 95–97 % at pH 7.5	
	Emulsion stability	80–96.9 %	
	Foaming capacity	80–133.3 % at pH 6	
	Viscosity	1.6–1.7 cP	
Sugar-beet leaf protein	Protein solubility	98.71 % at pH 7.5	Akyüz and Ersus (2021)
	Bulk density	80.73 kg/m^3^	
	Tapped density	99.77 kg/m^3^	
	Particle density	826.04 kg/m^3^	
	Carr Index	19.08 %	
	Hausner Ratio	1.24	
	Wettability	38.17 s	
	Dispersibility	77.90 %	
	Water activity	0.38	
*Raphanus sativus* L. leaf protein	Protein solubility	27.60–37.76 %	Kaur and Bhatia (2023)
	Water holding capacity	408 %	
	Oil holding capacity	335 %	
	Foaming capacity	14.9 % at pH 7.4	
	Foaming stability	20 %	
	Emulsion capacity	44.3 %	
	Emulsion stability	42.1 %	
	Protein solubility	37.76 % at pH 8	

### 6.1 Protein solubility

Protein solubility is closely linked to gelling, foaming, and emulsifying properties, ultimately guiding the applications of proteins in the food industry. From a thermodynamic perspective, protein solubility reflects the equilibrium between protein–protein and protein-solvent interactions under specific conditions and is directly associated with the physicochemical nature of the protein surface and indirectly reflects the alteration in the hydrophobicity, structure, and charge balance of the protein (Perović et al., 2020; Wei et al., 2024). Insoluble leaf proteins are typically bound to lipids and pigments to form photosynthetic complexes in thylakoid membranes or are associated with polysaccharides in the cell walls of some organisms. For soluble proteins, approximately 50 % of their composition is due to RuBisCo (Fatima et al., 2024). Solubility reaches its lowest point at the isoelectric point because this condition favours protein-protein interactions over protein-water interactions, leading to protein precipitation (Kumar et al., 2022). The ionic strength and pH of the solvent significantly influence protein solubility. Proteins exhibit the least solubility at their isoelectric point due to the absence of net charge, while alkaline or acidic solvents tend to enhance protein solubility. Additionally, proteins carry a net charge—either positive or negative—facilitating interactions between ionic groups in the solvent and charged amino acids on the protein surface. This promotes protein dispersion and solubilization (Navaf et al., 2023). The purity of leaf protein concentrate also plays a key role, as phytochemicals bound to leaf proteins can result in low protein solubility (Nynäs et al., 2023).

Generally, the properties of proteins are affected by treatments during processing and environmental conditions. EAE is observed to enhance solubility at alkaline pH. CLP and BLP notably showed maximum solubility of 29.4 mg/mL at pH 11 and 36.4 mg/mL at pH 10, respectively, with BLP exhibiting the highest solubility among cruciferous leaves (Sedlar et al., 2025). Similarly, the protein solubility profile for RLPC shows the lowest value at pH 4 (27.60 %) and the highest at pH 8 (37.76 %), indicating suitability for protein-rich carbonated beverages that function across a range of different pH levels (Kaur & Bhatia, 2023). In contrast, RLPC prepared by alkaline extraction exhibited maximum solubility at pH 12 (74 %), but decreased significantly at acidic pH levels (61 % at pH 2) (Kaur & Bhatia, 2022). The solubility of enzyme-extracted MLP varied depending on the isolation techniques. Isoelectric precipitation (IP) and ammonium sulphate precipitation (ASP) reached 97 % and 95 % solubility at pH 7.5, respectively, while a combined isoelectric-ammonium sulphate (IASP) method exhibited maximum solubility (94.4 %) at pH 8.5 (Ersus & Akyüz, 2023). These findings indicate that isolation conditions can influence solubility even when using the same leaf source.

Enzyme concentrations also significantly impacted solubility. In almond cake, the solubility of skim protein increased from 25.5 % to 48.2 % at pH 5 with an increase in enzymatic dosage from 0.15 % to 0.85 %. In contrast, alkaline-extracted skim protein showed a solubility value of around 19 %. This difference could be attributed to the enzymatic release of smaller and more soluble peptides (Souza et al., 2019). Compared to conventional methods, EAE under milder conditions produces proteins with better solubility. EAE avoids chemicals and is performed under mild conditions, producing high-quality proteins with improved solubility. The high solubility of hydrolysed proteins mainly results from the higher concentration of small peptides, which increase ionizable amino and carboxyl groups (Chandran et al., 2024). As Gao et al. (2024) stated, proteins with charged side chains have greater accessibility to solvents in aqueous solutions, enhancing their solubility. While increased concentrations of hydrophilic amino acids generally enhance protein solubility, aspartic acid, glutamic acid, and serine contribute more significantly to solubility than asparagine, glutamine, threonine, lysine, and arginine, particularly under conditions of high net charge. Among different leaf types, BLP showed the highest solubility, followed by CLP and RLPC, while MLP's solubility depended on the extraction method used.

### 6.2 Foaming properties

Foaming properties are influenced by factors such as solubility, surface hydrophobicity, the location of hydrophobic amino acid residues on the protein surface, presence of thiol groups, cations, anions, and protein composition (Jiang et al., 2021). For effective foaming capacity (FC), a protein molecule must exhibit strong surface activity by quickly adsorbing at the interface of two incompatible phases and reducing surface tension, as well as undergo conformational rearrangements and distribution at the interface. Conversely, for good foaming stability (FS), the protein should be capable of forming a viscoelastic film at the interface via intermolecular interactions (Li et al., 2019). FS affects the strength and gas permeability of protein films (Kaur & Bhatia, 2023) and is crucial for maintaining the textural and structural qualities in various products such as milk foams, cakes, and chocolate mousse (Dias & De Moura Bell, 2022).

EAE of MLP reported the highest FC at pH 6 for IP (133. 3 %), ASP (90. 9 %), and IASP (80 %). FC declined significantly at pH 4 for IP, ASP, and IASP, with values of 45. 5 %, 43. 8 %, and 45.5 %, respectively. This indicates that foaming attributes decrease around isoelectric points, where proteins are in a stable configuration. The isolation technique greatly influences the functional properties by affecting the surface activity of the protein, which alters its foaming stability. FS showed a similar trend, with pH 6 producing the most stable foams, and IP demonstrating high stability (116.7 % at 5 min and 83.3 % at 120 min). Therefore, both FC and FS are highly affected by the isolation method, with IP yielding the best results, followed by ASP and IASP (Ersus & Akyüz, 2023). FC is primarily influenced by protein solubility, peptide chain flexibility, and the number of hydrophobic groups, while FS mainly depends on the protein's ability to form a cohesive network at the interface through covalent and noncovalent interactions. Higher pH values can increase intermolecular repulsion, which is generally unfavourable for protein-protein interactions, leading to the degradation of FS (Wei et al., 2024). Although enzyme-extracted MLP exhibited excellent foaming properties, RLPC showed poor results. FC and FS values were relatively low, at 14.9 % (pH 7.4) and 20 %, respectively, as reported by Kaur and Bhatia (2023), making them unsuitable as foaming and whipping agents in food products such as bakery items, drinks, and ice creams. Even alkaline-extracted RLPC showed only marginal improvement, with FC and FS values of 20.4 % (pH 7.4) and 34 %, respectively (Kaur & Bhatia, 2022). The findings indicate that MLP clearly outperformed RLPC, with IP being the most effective isolation method for preserving FC and FS. pH and protein composition significantly influence FC. Additionally, the foaming potential of the protein may be limited by its compact structure, which has low interface absorption and is generally difficult to unfold (Navaf et al., 2023).

### 6.3 Emulsifying properties

The emulsifying property is a significant interfacial characteristic of proteins. It refers to their ability to interact with and stabilise oil–water mixtures, thereby preventing phase separation (Wei et al., 2024). When the protein enters the oil-water interface, it unfolds gradually. The hydrophobic part orients towards the oil, and the hydrophilic part towards the water, forming a film around the droplets, reducing interfacial tension, and leading to emulsion formation (Navaf et al., 2023). Generally, a good emulsifier should demonstrate a balance between polar and non-polar groups to have effective surface activity and achieve sufficient water solubility. This balance significantly influences the physical and oxidative stability of emulsions. Changes in environmental pH modify the net charge and conformation of proteins at the interface, thereby impacting their emulsifying capacity. In addition to pH, enzymatic hydrolysis of proteins enhances their solubility and emulsification capacity as a result of alterations in their globular structure (Padial-Domínguez et al., 2020). Importantly, protein content does not necessarily correlate with protein function attributes such as emulsification. For example, lupin has a higher protein content than wheat or green peas, but it is not considered a good emulsifier (Pathania et al., 2018). Emulsifying properties are essential for the production and stabilisation of emulsions in food products like dressings, beverages, and sauces, and are linked to protein characteristics such as amino acid composition, structure, surface hydrophobicity, and molecular weight (Dias & De Moura Bell, 2022; Ortega et al., 2024).

Ersus and Akyüz (2023) reported the lowest EC values for EAE of MLP at the isoelectric point, as IP (38.4 %), ASP (42.9 %), and IASP (41.7 %), while the highest values were observed between pH 6 and pH 8. Notably, IP produced smaller hydrophobic subunits, promoting strong interactions with lipids, while ASP preserved the structural integrity of proteins and resulted in more stable emulsions at alkaline pH. Emulsion stability (ES) was highest at pH 6 for both IP and IASP, whereas ASP showed the highest stability at pH 8. Thus, the protein isolation technique significantly influences emulsifying properties, with IP showing the highest EC values, followed by IASP and ASP, although ASP formed more stable emulsions at alkaline pH. Oil-water interfacial reactions generally involve high protein participation due to polypeptide chain unfolding under alkaline conditions. For enzyme-extracted RLPC, emulsion capacity (44.3 %) and emulsion stability (42.1 %) were suitable for use as functional additives to stabilise food emulsions (Kaur & Bhatia, 2023), while alkaline extraction yielded slightly higher EC and ES values at 48.1 % and 47.8 %, respectively (Kaur & Bhatia, 2022). Overall, this indicates that both the leaf source and extraction techniques are key factors in determining emulsifying properties for food applications.

### 6.4 Water activity

The water activity for MLP and SLP was observed to be 0.30 and 0.38, respectively. Since the values are below 0.65, microbial growth is likely to be limited, ensuring the long-term storage stability of protein concentrates (Akyüz & Ersus, 2021; Ersus & Akyüz, 2023). MLP, comparatively, showed slightly lower water activity, indicating greater microbial stability and potentially a longer shelf life than SLP. Water activity is related to the hydrophobicity of the amino acids in the protein structure. As mentioned above, polar amino acids are hydrophilic, while nonpolar amino acids are hydrophobic. Because the reported values are low, it suggests that hydrophilic amino acids are present in smaller amounts compared to hydrophobic ones.

### 6.5 Gelation

Gelation is the ability of proteins to change from a sol (fluid) to a gel (semi-solid) state. This involves the physical or chemical crosslinking of protein molecules, leading to the formation of a continuous network that can trap and bind water molecules (Hadidi et al., 2024). In other words, protein denaturation followed by network formation is the process that triggers gelation, which is a functional property that can significantly impact the texture of food products (Grácio et al., 2023). The minimum ‘least gelation concentration’ for RLPC was noted to be 7 % (*v*/v) and demonstrated superior gelation properties, making it a potential additive in food materials for gel formation (Kaur & Bhatia, 2023).

### 6.6 Water (WHC) and oil holding capacity (OHC)

Limited studies are available on enzyme-extracted leaf proteins and their functionality. Water holding capacity (WHC) and oil holding capacity (OHC) refer to the amount of water or oil that a unit weight of protein can hold. These functional properties are important for preventing the loss of liquid from products during processing and storage, which directly affects the texture and flavour of food materials. Moreover, OHC plays a significant role in flavour retention in food products because fats are mainly bound by the non-polar side chains of proteins (Wei et al., 2024). The amount of water associated with proteins is closely related to their amino acid profile and increases with the number of charged residues (Dias & De Moura Bell, 2022). As reviewed by Navaf et al. (2023), a significant amount of water interacts with proteins through hydrophilic groups present in the side chain of the protein via hydrogen bonding. Therefore, polar amino acids in proteins contribute to the WHC value. Other factors affecting WHC include pH, protein composition and conformation, ionic strength, and protein concentration. In contrast, OHC is mainly influenced by the particle size, charge distribution, temperature and amino acids interaction. For enzyme-extracted RLPC, the oil holding capacity was 335 %, while the water holding capacity was estimated at 408 %. The protein content of RLPC is predicted to contain more protein side chains with hydrophobic groups, resulting in the high OHC value (Kaur & Bhatia, 2023). Conversely, alkaline-extracted RLPC showed a WHC of 352 % and OHC of 280 % (Kaur & Bhatia, 2022). The improved OHC of enzyme-extracted proteins suggests enhanced flavour-binding potential, while higher WHC promotes better moisture retention in processed foods. Various interactions—including hydrophobic interactions, dipole-dipole interactions, ion-dipole interactions, and dipole-induced dipole interactions—contribute to the water binding process. Additionally, the structure of proteins, particularly pore size, affects water–protein interactions. High water absorption capacity is essential for reducing moisture loss in packaged baked goods, thereby preserving their mouthfeel, texture, and freshness (Chandran et al., 2024). Overall, available studies indicate that EAE provides better WHC and OHC values than alkaline extraction; however, further comparative research with different leaf sources is necessary to understand the trend.

## 7 Potential industrial applications of leaf protein and market trends

For the future, green leaf proteins are recognised by the United Nations Food and Agriculture Organisation (FAO) as a major source of food proteins because they are abundant in nature, highly nutritious, and sustainable (Hadidi et al., 2024). Dietary patterns worldwide are undergoing a major shift due to increasing health awareness and sustainability concerns. The vegetarian population in Italy has risen by 94.4 % (2011–2016), according to The Vegan Society. In 2017, 51 % of Danes reportedly did not consume meat one day a week, and this number notably increased in 2019, with 30 % saying they eat far less meat and have reduced their consumption within the last five years (Aschemann-Witzel et al., 2021). These trends indicate a gradual decline in reliance on animal proteins in favour of plant-based alternatives. A cohort study by Zhang et al. (2022), conducted in North China on 14,541 subjects, reported that replacing animal protein processed foods with plant proteins is the most effective way of reducing non-alcoholic fatty liver disease (NAFLD). Javed et al. (2023) incorporated *Moringa oleifera* leaf protein into the basal diets of rats, which increased Hb levels and other haematological indices. Diet supplementation also reduced liver enzyme levels and showed a hepatoprotective effect. As health consciousness increases, a significant shift in nutritional preference from animal to plant proteins is evident. Many researchers are exploring leaf proteins, as they are comparatively better substitutes for animal and legume proteins in terms of availability, cost-effectiveness, and renewability. For example, Kaur and Bhatia (2023) reported high mineral content in RLPC, including zinc, copper, manganese, chromium, iron, calcium, potassium, magnesium, and phosphorus. Additionally, the concentrate showed significant antioxidant activity in ferric reducing antioxidant activity (FRAP) (25.88 %), DPPH (28.66 %), and ABTS (83.29 %) assays, while microbial load remained within acceptable limits. The functional properties of various enzyme-extracted leaf proteins have already been discussed above; more products, such as energy bars, patties, and vegan cookies, can be developed and commercialised. Ducrocq et al. (2020) chose the traditional approach of enriching food products with plant proteins to boost protein content and provide a balanced amino acid profile. RuBisCO-enriched wheat dough was prepared, and RuBisCO was found to maintain the dough's elastic potential better than gluten and pea protein. Also, the study highlighted the potential ability of RuBisCo to increase the plant protein content of cereal-based foods.

Commercial interest in leaf proteins has increased in recent years. Anoop et al. (2023) reviewed several companies incorporating leaf proteins as food ingredients. For example, the Netherlands-based Rubisco startup extracts alfalfa and water lentil proteins to formulate protein gels and powders. Royal Cosun, the parent company of Suiker Unie in the Netherlands, holds a patent for sugar beet leaf protein extraction. These proteins can be used in the production of meat substitutes, beverages, cakes, desserts, and sauces. Leaf Foods claims its extracted protein is odourless and tasteless, facilitating its incorporation into traditional plant protein sources. Other companies engaged in leaf protein development include The Leaf Protein Company (Australia) and NIZO (Netherlands). Plantible has introduced a plant-based protein, Rubi protein, which is reported to improve taste and texture in various food applications (Plantible, accessed 2025). Rubisco Foods is a Dutch company specialising in the development and production of plant-based food and feed ingredients, particularly water lentil and alfalfa protein powders and fibres (Rubisco Foods, accessed, 2025). Luzerne Recherche et Développement, a French company, has produced a concentrated source of *Medicago Sativa* (Luzixine™)*,* which is recognised as a novel food by the European Commission. The concentrate is considered ideal for the formulation of nutraceutical and natural health products (Luzixine™, accessed 2025). Another initiative to upcycle the discarded agricultural biomass is taken by Day8, a company working to create sustainable, high-quality Rubisco protein (Day 8, accessed 2025). Currently, leaf protein applications include bakery products, meat analogues, and novel delivery systems. However, industrial challenges persist. Large-scale production is limited by low extraction yields, the presence of antinutritional components, and difficulties in maintaining protein functionality after processing. Enzyme-assisted extraction, although promising, faces challenges related to cost and recovery efficiency. Most research remains at an early stage, and broader commercialisation will require optimisation of extraction methods, validation of safety and bio functionality, and consumer acceptance studies.

## 8 Future prospective

EAE offers significant potential for eco-friendly extraction of plant proteins, especially from underused resources like green leaves. Future research should focus on optimising enzyme formulations tailored for different plant matrices, including enzyme mixtures that effectively break down complex cell wall components. Developing affordable, customised enzymes can address issues of high costs and scalability, making EAE more practical for industrial applications. Advances in biotechnology, such as enzyme engineering and immobilisation techniques, can enhance enzyme stability and reusability, reducing processing costs and environmental impact. Additionally, efforts to lower anti-nutritional factors and improve the bioavailability of extracted proteins are crucial for expanding their use in food, feed, and nutraceutical sectors. Valorising agricultural by-products like waste leaves can promote circular bioeconomy practices, reducing food waste and pollution. Scaling up EAE for industrial use also requires research into process design, energy efficiency, and yield optimisation. Lastly, exploring the use of extracted proteins in developing innovative products, such as meat substitutes, functional drinks, and protein isolates, will meet increasing consumer demand for sustainable, plant-based proteins.

## 9 Conclusion

EAE has an emerging technique for extracting plant-based proteins, addressing the increasing global demand for alternative protein sources. This review highlights the advantages of EAE, including higher protein content, improved functional properties, and a smaller environmental footprint compared to conventional methods. By breaking down plant cell walls and releasing bound proteins under mild conditions, EAE preserves protein quality while reducing energy consumption and chemical use. EAE shows excellent potential for valorising underused green leaves and agro-industrial by-products, supporting the principles of a circular bioeconomy and minimising food waste. Additionally, EAE's ability to extract proteins with superior techno-functional characteristics, such as solubility, emulsification, and foaming, makes it particularly valuable for applications in food, nutraceuticals, and animal feeds. However, challenges remain, including high enzyme costs, process scalability, and the limited bioavailability of proteins from leaves. Future research should focus on improving enzyme formulations, integrating EAE with advanced green technologies, and developing cost-effective methods for large-scale industrial production. Addressing these issues could enable EAE to revolutionise protein extraction, offering sustainable solutions to meet global nutritional needs while promoting environmental sustainability and innovation in developing protein-based products.

## CRediT authorship contribution statement

**Ankita Sharma:** Writing – original draft. **Shalini Sharma:** Writing – review & editing, Supervision. **Godasritha Ramaraju:** Writing – review & editing, Supervision. **Prasad Rasane:** Resources, Formal analysis. **Sezai Ercisli:** Validation, Software. **Jyoti Singh:** Conceptualization.

## Ethical approval

Not applicable.

## Declaration of competing interest

The authors declare that they have no known competing financial interests or personal relationships that could have appeared to influence the work reported in this paper.

## Data Availability

Data will be made available on request.
